# Management of persistent residual growth of high-grade glioma: a case report highlighting clinical and therapeutic challenges

**DOI:** 10.1093/omcr/omaf198

**Published:** 2025-11-26

**Authors:** Masab Ali, Muhammad Hassan, Muhammad Husnain Ahmad, Ilsa Babar, Sana Javeriya, Muhammad Asfand Nadeem, Humza Saeed

**Affiliations:** Department of Medicine, Punjab Medical College, Sargodha Road, District Faisalabad, Punjab 38000, Pakistan; Department of Medicine, Punjab Medical College, Sargodha Road, District Faisalabad, Punjab 38000, Pakistan; Department of Medicine, Tentishev Satkynbai Memorial Asian Medical Institute, Gagarina 58, Kant, Chuy 725000, Kyrgyzstan; Department of Medicine, King Edward Medical University, Neelagumbad, Anarkali, Lahore, Punjab 54000, Pakistan; Department of Medicine, JIIU's Indian Institute of Medical Science and Research (IIMSR), Aurangabad-Jalna Road, Warudi, Taluka Badnapur, Jalna 431202, Maharashtra, India; Department of Medicine, King Edward Medical University, Neelagumbad, Anarkali, Lahore, Punjab 54000, Pakistan; Department of Medicine (Medical Unit - Internal Medicine), District Headquarters (DHQ), Hospital Kashmiri Bazaar Road, Raja Bazar, Rawalpindi, Punjab 46000, Pakistan

**Keywords:** glioma, headache, synaptophysin, seizures, craniotomy

## Abstract

**Introduction:**

Gliomas, particularly glioblastomas (GBMs), are the most common malignant primary brain tumors, comprising nearly 80% of CNS malignancies. Despite aggressive treatment, recurrence is common due to its infiltrative nature and therapy resistance, with median survival around 14–15 months.

**Case Presentation:**

A 48-year-old male with a history of WHO Grade IV glioblastoma presented five years after subtotal resection with recurrent symptoms. He had not received adjuvant radiotherapy and was lost to follow-up. Recent imaging showed a lesion near the original tumor site. He underwent repeat craniotomy with subtotal excision. The patient’s neurological status remained stable postoperatively, although headaches persisted.

**Discussion:**

Recurrent GBMs pose significant management challenges due to diagnostic complexity and limited treatment options. Multimodal imaging and individualized strategies, including surgery and potential adjuvant therapies, are critical.

**Conclusion:**

This case underscores the need for vigilant follow-up and a personalized, multidisciplinary approach to improve outcomes in patients with recurrent glioblastoma.

## Introduction

Gliomas comprise a heterogeneous group of primary central nervous system (CNS) tumors, classified by histopathologic and molecular features that reflect their presumed cell of origin. This category includes astrocytic tumors—such as diffuse astrocytoma, anaplastic astrocytoma, and glioblastoma (GBM)—as well as oligodendrogliomas, ependymomas, and mixed lineage gliomas [1]. Collectively, gliomas account for approximately 80% of all malignant primary brain tumors, rendering them the most prevalent malignant neoplasms of the CNS [2].

High-grade gliomas, particularly GBM (WHO Grade IV), are typically managed with a multimodal treatment paradigm consisting of maximal safe surgical resection followed by adjuvant radiotherapy and temozolomide-based chemotherapy. However, despite these aggressive interventions, glioblastomas are notorious for their diffuse infiltration, rapid progression, and resistance to standard therapies. Consequently, disease recurrence is virtually inevitable, and the median overall survival remains limited to 14–15 months post-diagnosis [3]. Clinically, glioblastoma often presents with a short interval of progressive neurological decline, occasionally mimicking acute cerebrovascular events. Patients may exhibit focal neurological deficits, cognitive dysfunction secondary to parenchymal necrosis, signs of elevated intracranial pressure, intractable headaches due to mass effect, and seizures. These symptoms vary depending on tumor location and are often nonspecific, complicating early diagnosis and necessitating prompt neuroimaging for accurate assessment [4].

Radiographically, recurrence most commonly manifests within 2–3 cm of the initial resection margin and is best identified through contrast-enhanced MRI. The management of recurrent or progressive GBM poses significant therapeutic challenges due to the paucity of curative options and the tumor’s continued evolution. Salvage strategies may include repeat surgical resection for cytoreduction and symptom control, re-irradiation in carefully selected cases, systemic therapies such as temozolomide rechallenge or bevacizumab [5], and participation in clinical trials. Treatment selection is highly individualized, taking into account factors such as tumor location, prior treatment history, patient performance status, and the interval to recurrence. Systemic therapies in the recurrent setting are largely palliative, with no agent demonstrating unequivocal survival benefit. Alkylating agents, particularly nitrosoureas such as lomustine (CCNU) and fotemustine, remain commonly employed. Lomustine is widely utilized and has served as the control arm in several landmark trials. Fotemustine, which effectively penetrates the blood–brain barrier, may offer therapeutic value in select patients. Current European Association of Neuro-Oncology (EANO) guidelines recognize both agents as reasonable options for recurrent or progressive disease, highlighting their relevance within multidisciplinary treatment frameworks [6].

Despite the availability of systemic and surgical options, the management of recurrent glioblastoma remains predominantly palliative, with an emphasis on maintaining neurological function and quality of life. These limitations underscore the urgent need for innovative, personalized therapeutic approaches aimed at improving long-term outcomes in this devastating disease.

**Figure 1:**
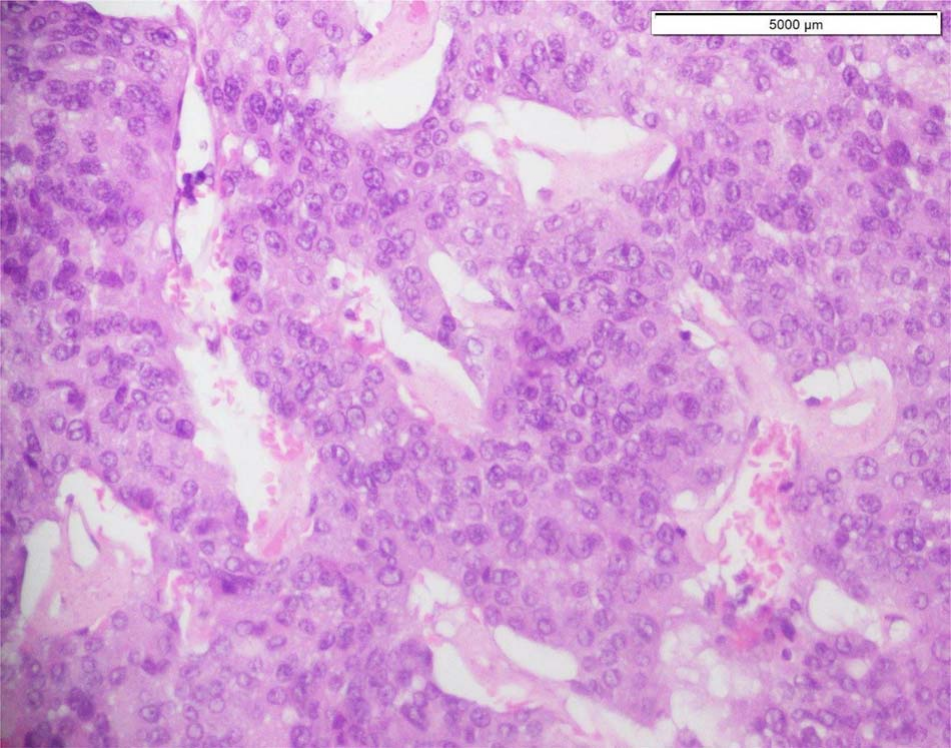
Histological section showing clusters of densely packed embryonal cells with minimal cytoplasm, indicative of high cellularity characteristic of recurrent high-grade glioma.

## Case presentation

A 48-year-old male presented with progressive neurological symptoms suggestive of tumor residual growth five years following initial treatment for glioblastoma. At the time of his first diagnosis, he underwent a craniotomy with subtotal resection of a lesion that was histopathologically confirmed as a WHO Grade IV glioblastoma. Although standard-of-care management includes postoperative radiotherapy, the patient voluntarily left the hospital against medical advice following surgery and consequently did not receive adjuvant treatment. He was subsequently lost to follow-up until his recent presentation.

**Figure 2:**
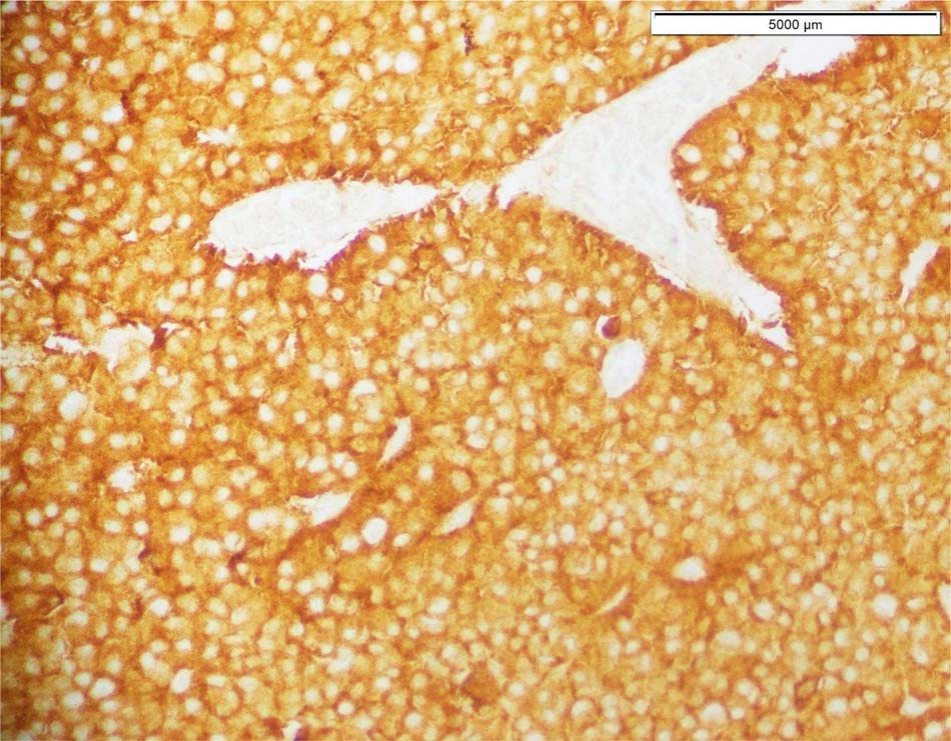
Immunohistochemical staining demonstrating strong positivity for synaptophysin, confirming the neuronal differentiation typical of high-grade embryonal tumors.

Histopathological analysis of the initial tumor revealed a high-grade glioma characterized by marked nuclear atypia, cellular pleomorphism, and microvascular proliferation, hallmarks of glioblastoma (Fig. 1). Immunohistochemical staining demonstrated strong synaptophysin positivity (Fig. 2). However, molecular testing for key prognostic and diagnostic markers including IDH1/IDH2 mutations, MGMT promoter methylation, and ATRX expression was not performed due to limited institutional access to advanced molecular diagnostics. Despite this limitation, these markers are now integral to glioma classification, prognostication, and therapeutic stratification.

Following the initial surgery, the patient remained clinically stable, with no radiological evidence of recurrence on serial imaging. However, in the weeks preceding his current admission, he developed progressively worsening headaches, episodic confusion, and memory disturbances. There was no antecedent history of trauma, infection, or systemic illness. On examination, he was alert and oriented, with a Glasgow Coma Scale (GCS) score of E4V5M6. Vital signs were stable, and he was afebrile. Neurological examination did not reveal new focal deficits, although he reported persistent, moderate-to-severe headaches.

The differential diagnosis included true tumor recurrence, radiation necrosis, pseudo-progression, and, less commonly, infectious or metabolic encephalopathies. Given the absence of prior radiotherapy, pseudo-progression and radiation necrosis were considered unlikely. Infectious and metabolic causes were also excluded based on the lack of systemic signs, laboratory abnormalities, or fever. Ultimately, the combination of progressive neurological symptoms and neuroimaging—demonstrating a contrast-enhancing lesion with mass effect in the original tumor bed—strongly supported the diagnosis of recurrent glioblastoma.

**Figure 3:**
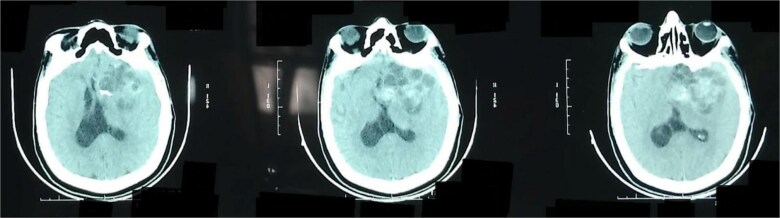
CT scan revealing a recurrent lesion in the right frontotemporal lobe, extending to the suprasellar region. The space-occupying lesion exerts a mass effect on both the lateral and third ventricles, contributing to ventricular compression.

Cross-sectional imaging, including CT and contrast-enhanced MRI, revealed a lesion within 2–3 cm of the original tumor site, consistent with the typical spatial pattern of residual growth of glioblastoma (Fig. 3). The lesion demonstrated imaging characteristics concerning active tumor regrowth. Although PET imaging can provide superior discrimination between tumor recurrence and post-treatment changes, it was not performed due to limited institutional access and the need for prompt intervention, given the patient’s neurological decline. Consequently, the diagnosis was established based on clinical history, imaging morphology, and recurrence patterns.

Given the lesion’s associated mass effect and the patient’s worsening symptoms, non-surgical options such as stereotactic radiosurgery (SRS) and systemic therapy were deemed suboptimal. SRS was not suitable due to the urgency of symptom relief, and systemic therapies were considered inadequate for immediate decompression. Thus, repeat surgical resection was pursued as the most appropriate intervention to alleviate intracranial pressure, mitigate mass effect, and facilitate subsequent adjuvant therapy. The risks of surgery, such as potential morbidity and subtotal resection, were weighed against the benefits of symptomatic relief and creating a favorable environment for postoperative adjuvant therapy.

Repeat craniotomy with subtotal tumor resection was performed under general anesthesia by an experienced neurosurgical team. The decision to opt for subtotal resection was made to avoid compromising critical brain structures, ensuring the balance between tumor removal and neurological function preservation. Preoperative optimization included analgesia and anticonvulsants to manage symptoms. Postoperatively, the patient was transferred to the intensive care unit (ICU) for close monitoring, with an emphasis on pain control and prevention of complications. Although postoperative MRI images were not available for direct review, the radiology report described imaging performed within 48 h of surgery. It documented a residual contrast-enhancing lesion adjacent to the resection cavity measuring approximately 1.2 × 1.5 cm, with markedly reduced mass effect relative to the preoperative state. No evidence of acute hemorrhage, midline shift, or ischemic changes was noted. Ventricular configuration was preserved, and the overall findings were consistent with successful surgical decompression.

On the first postoperative day, the patient’s neurological status remained stable, with a GCS of E4V5M6. He was alert, following commands, and hemodynamically stable. Oral intake was adequate. Although he continued to report headaches, these were managed effectively with analgesics. No new focal neurological deficits were observed, and he remained under close neurosurgical observation. Although a formal pathological analysis of the resected tissue was not conducted following the repeat craniotomy, the diagnosis of recurrence was supported by characteristic radiological features, the tumor’s anatomical location, and the patient’s prior history of WHO Grade IV glioblastoma. At the time of surgery, there were institutional constraints, including limited access to advanced histopathological processing facilities, and the urgency of symptom management also precluded complete pathological confirmation.

A multidisciplinary postoperative management plan was initiated in accordance with contemporary standards for recurrent high-grade gliomas. Given the patient’s preserved functional status and the absence of prior adjuvant therapy, the therapeutic strategy aimed to achieve maximal tumor control while minimizing treatment-related morbidity. The patient was deemed an appropriate candidate for primary external beam radiotherapy, with a standard protocol of 60 Gy in 30 fractions over six weeks. This was expected to enhance local tumor control and delay disease progression. Concurrently, adjuvant chemotherapy with temozolomide (TMZ) was initiated at a dose of 150–200 mg/m^2^/day for five consecutive days in 28-day cycles, contingent on hematologic tolerance and clinical response. Supportive measures included corticosteroids to reduce cerebral edema, anticonvulsants for seizure prophylaxis, and neurocognitive monitoring. The patient was enrolled in a structured follow-up program incorporating serial imaging and multidisciplinary evaluations to guide further therapeutic decisions, with a sustained focus on balancing oncologic efficacy and neurological function preservation.

## Discussion

The WHO categorizes gliomas, a type of brain tumor, into four grades based on their cellular characteristics—with grades I and II considered low-grade gliomas (LGGs) and grades III and IV categorized as HGGs [7]. HGGs account for the majority of primary brain tumors in adults and come with a challenging prognosis. Primary malignant brain tumors are identified according to their tissue characteristics as anaplastic gliomas (including anaplastic astrocytoma, anaplastic oligodendroglioma, and anaplastic oligoastrocytoma; classified as WHO grade III) and glioblastomas (GBMs; WHO grade IV), with GBMs making up about 60–70% of these cases [8]. The recurrence rate of HGGs is high, with GBM patients having an estimated survival time of 25–30 weeks post-disease progression and those with anaplastic gliomas having a slightly longer estimate of 39–47 weeks post-progression [9, 10]. Clinically, GBM often presents with a rapidly progressive course, with the majority of patients exhibiting symptoms within a three-month window prior to diagnosis [11]. Standard presenting features include headaches, cognitive decline, seizures, and focal neurological deficits—primarily driven by increased intracranial pressure and tumor location [12]. Notably, only 10–40% of patients with brain tumors like glioblastoma present with focal neurological deficits [13]. Neuroimaging plays a pivotal role in both diagnosis and surveillance. While contrast-enhanced CT and MRI remain the standard modalities for initial tumor identification and characterization, distinguishing true tumor recurrence from post-treatment changes such as radiation necrosis remains challenging. Advanced imaging techniques, including positron emission tomography (PET) and single-photon emission computed tomography (SPECT), can provide additional insight into tumor metabolism and cellular viability. GBM recurrence most commonly occurs within 2–3 cm of the original lesion and typically manifests around 32–36 weeks post-treatment [14]. Several prognostic factors influence therapeutic decision-making in recurrent GBM, including performance status, age, molecular profile, and extent of previous treatment [15]. While treatment paradigms for newly diagnosed GBM are well established—incorporating maximal safe resection followed by concurrent radiotherapy and temozolomide chemotherapy—management of recurrent disease remains a clinical challenge characterized by limited therapeutic efficacy and high relapse rates [12].

Surgical re-intervention plays a vital role in select patients with recurrent GBM, particularly in those experiencing neurological deterioration due to mass effect or in whom histopathological confirmation is necessary to distinguish recurrence from treatment-related changes [8]. Surgical approaches may include gross total or subtotal resection, stereotactic biopsy, or open biopsy in patients who decline more extensive procedures. Locoregional therapies, such as laser interstitial thermal therapy (LITT), and supportive measures are also integrated into the multidisciplinary treatment armamentarium [16]. The utility of re-irradiation is constrained by prior dosing and the risk of radiation-induced toxicity to adjacent healthy brain parenchyma, especially when the interval between treatments is short [8]. Chemotherapy offers modest benefits in recurrent settings, with temozolomide (TMZ) remaining a cornerstone. Interestingly, TMZ was initially introduced for recurrent gliomas and later adopted as first-line therapy [8]. In selected patients, rechallenge with TMZ may be beneficial, particularly in those with a long progression-free interval and favorable molecular profiles [17].

Given the high vascular density and overexpression of vascular endothelial growth factor (VEGF) in GBM, anti-angiogenic therapy with agents such as bevacizumab has demonstrated efficacy in symptom management and radiographic response, though survival benefits remain limited [18]. Dexamethasone is routinely employed to manage neurological deficits, headaches, and drowsiness, which are indicative of increased intracranial pressure [17]. In some European countries, nitrosourea-based agents such as fotemustine continue to be incorporated into individualized regimens [17]. Tumor treating fields (TTF), a non-invasive modality employing alternating electric fields to disrupt mitotic spindle formation, has gained approval as an adjunctive treatment in newly diagnosed and recurrent GBM. Its incorporation into clinical practice is increasing, particularly given its favorable safety profile [19].

Emerging adjunctive therapies are under active investigation. These include precision radiation modalities such as tomotherapy, biophysical techniques like electro-hyperthermia, and biologic approaches such as oncolytic virotherapy. The combination of virotherapy and immunotherapy, specifically oncolytic viruses and cancer vaccines, has garnered growing interest because of its demonstrated synergistic immune activation and tumor regression in preclinical studies and early-phase clinical trials [20]. Nanomedicine represents a particularly promising frontier in GBM therapeutics. Nanocarrier platforms designed to traverse the blood–brain barrier (BBB) enhance intratumoral drug delivery while minimizing systemic toxicity. NanoTherm™, the first clinically approved nanoparticle-based therapy for GBM, utilizes magnetic nanoparticles for localized hyperthermia and has shown clinical feasibility. However, widespread application is hindered by challenges related to manufacturing scalability, regulatory oversight, and cost-effectiveness [21]. Molecular advances have also catalyzed the development of targeted therapies, including small molecule inhibitors and monoclonal antibodies aimed at dysregulated oncogenic pathways and tumor-specific surface markers. However, treatment resistance remains a significant obstacle, driven by tumor heterogeneity, the presence of glioma stem-like cells (GSCs), and restricted drug delivery across the BBB. GSCs, in particular, contribute to tumor recurrence and therapeutic resistance, highlighting their importance as a future therapeutic target [22]. Immunotherapy has emerged as a potential paradigm-shifting modality in GBM treatment. While durable responses have been limited to a subset of patients, the integration of molecular and immune biomarkers into treatment planning may enable more personalized therapeutic approaches. Stratifying patients based on immunogenomic profiles can enhance predictive accuracy and optimize clinical outcomes [23]. Among investigational immunotherapies, chimeric antigen receptor (CAR) T-cell therapy holds promise. However, its translation into GBM care is impeded by CNS immune privilege, antigen heterogeneity, and a profoundly immunosuppressive tumor microenvironment (TME). Innovative strategies—including SynNotch CAR-T systems and the use of low-intensity focused ultrasound to transiently disrupt the BBB—are under preclinical and early clinical evaluation to improve T-cell infiltration and specificity [24].

In conclusion, although the current standard of care for glioblastoma—including surgical resection followed by concurrent chemoradiation—offers limited survival benefits, a growing armamentarium of innovative therapies is under development. These include nanotechnologies, immunotherapeutic platforms, and combination regimens that aim to overcome the inherent biological complexities of GBM. Continued translational and clinical research is imperative to validate and integrate these emerging strategies into multimodal treatment paradigms. In the context of our patient, the recurrence of glioblastoma five years after subtotal resection in the absence of adjuvant chemoradiotherapy represents an atypical clinical course. The patient was lost to follow-up postoperatively and did not receive standard adjuvant treatment. Upon symptomatic recurrence characterized by mass effect and progressive neurological deterioration, prompt surgical intervention was prioritized per current guidelines advocating for decompressive resection in symptomatic patients. Stereotactic radiosurgery was considered but ultimately deemed unsuitable given the urgency of clinical intervention. Postoperatively, the patient was initiated on external beam radiotherapy and adjuvant temozolomide, aligned with standard first-line regimens for newly diagnosed GBM, given the lack of prior exposure and preserved functional status.

This case underscores the need for flexible, individualized treatment strategies in recurrent GBM, particularly in settings where standard treatment trajectories are disrupted. It further highlights the importance of early diagnosis, timely intervention, and a multidisciplinary, resource-sensitive approach. As the therapeutic landscape continues to evolve, the integration of novel and personalized therapies will be essential to improving outcomes and quality of life in patients with this devastating malignancy.

## Conclusion

The conclusions drawn from this case and the relevant literature highlight that, while treatments for newly diagnosed GBM are well-established, the therapeutic options for recurrent GBMs remain less clearly defined. A multimodal approach—comprising surgery, targeted therapies, and potentially re-irradiation—is recommended, emphasizing the need to balance the potential benefits with the risks of adverse effects, given the aggressive nature of GBMs and their high likelihood of recurrence.

## Data Availability

Data and materials are available upon request from the corresponding author.
